# Detecting QT prolongation from a single-lead ECG with deep learning

**DOI:** 10.1371/journal.pdig.0000539

**Published:** 2024-06-25

**Authors:** Ridwan Alam, Aaron Aguirre, Collin M. Stultz

**Affiliations:** 1 Research Laboratory of Electronics, Massachusetts Institute of Technology, Cambridge, Massachusetts, United States of America; 2 Computer Science & Artificial Intelligence Laboratory, Massachusetts Institute of Technology, Cambridge, Massachusetts, United States of America; 3 Department of Electrical Engineering and Computer Science, Massachusetts Institute of Technology, Cambridge, Massachusetts, United States of America; 4 Wellman Center for Photomedicine, Massachusetts General Hospital, Boston, Massachusetts, United States of America; 5 Division of Cardiology, Massachusetts General Hospital, Boston, Massachusetts, United States of America; 6 Harvard Medical School, Boston, Massachusetts, United States of America; 7 Harvard-MIT Program in Health Sciences and Technology, Cambridge, Massachusetts, United States of America; 8 Institute for Medical Engineering and Science, Massachusetts Institute of Technology, Cambridge, Massachusetts, United States of America; Columbia University, UNITED STATES

## Abstract

For a number of antiarrhythmics, drug loading requires a 3-day hospitalization with continuous monitoring for QT-prolongation. Automated QT monitoring with wearable ECG monitors would enable out-of-hospital care. We therefore develop a deep learning model that infers QT intervals from ECG Lead-I—the lead that is often available in ambulatory ECG monitors—and use this model to detect clinically meaningful QT-prolongation episodes during Dofetilide drug loading. QTNet–a deep neural network that infers QT intervals from Lead-I ECG–was trained using over 3 million ECGs from 653 thousand patients at the Massachusetts General Hospital and tested on an internal-test set consisting of 633 thousand ECGs from 135 thousand patients. QTNet is further evaluated on an external-validation set containing 3.1 million ECGs from 667 thousand patients at another healthcare institution. On both evaluations, the model achieves mean absolute errors of 12.63ms (internal-test) and 12.30ms (external-validation) for estimating absolute QT intervals. The associated Pearson correlation coefficients are 0.91 (internal-test) and 0.92 (external-validation). Finally, QTNet was used to detect Dofetilide-induced QT prolongation in a publicly available database (ECGRDVQ-dataset) containing ECGs from subjects enrolled in a clinical trial evaluating the effects of antiarrhythmic drugs. QTNet detects Dofetilide-induced QTc prolongation with 87% sensitivity and 77% specificity. The negative predictive value of the model is greater than 95% when the pre-test probability of drug-induced QTc prolongation is below 25%. These results show that drug-induced QT prolongation risk can be tracked from ECG Lead-I using deep learning.

## Author summary

Ambulatory ECG monitoring, coupled with sophisticated algorithms for identifying instances of QT prolongation, can enable outpatient Dofetilide-loading in low-risk patients. As most ambulatory ECG monitors record the equivalent of a Lead-I ECG, we developed a model, QTNet, that can estimate QT intervals using ECG Lead-I alone. QTNet estimates QT intervals that are similar to those generated from the 12-lead ECG by the clinical ECG machines, with a mean absolute error of 12ms and a Pearson correlation coefficient of 0.91. When applied in a zero-shot manner (without any fine-tuning) to an external population undergoing Dofetilide loading, QTNet identifies when clinically critical QT prolongation occurs during drug loading. QTNet is a novel regression model that can be used on Lead-I ECG streams, potentially from wearable devices at out-of-hospital settings, for health critical applications such as drug-induced QT prolongation tracking.

## Introduction

In addition to stroke prophylaxis, therapeutic options in patients with atrial fibrillation (AF) involve either rate control or rhythm control [1,2]. While rhythm control is preferred in patients who cannot tolerate AF, recent data suggests that some patients may benefit from early interventions that reduce AF burden and maintain normal sinus rhythm. This suggests that the maintenance of normal sinus rhythm may be beneficial in patients who do not have symptoms associated with AF [3–5].

A number of antiarrhythmic drugs have proven efficacious with respect to maintaining normal sinus rhythm and shortening the duration and symptoms associated with AF [6–8]. As many of these agents have important side effects, however, the initiation of these agents is typically done under the supervision of trained personnel in an inpatient setting. QT prolongation–an adverse side effect of many antiarrhythmic drugs–increases the risk of polymorphic ventricular tachycardia in the form of torsade de pointes and, consequently, administration of antiarrhythmic therapy is often done in an inpatient setting. Indeed, the concern for significant QT prolongation necessitates inpatient observation, continuous telemetry, and review of 12-lead ECGs before and after each drug dose for many antiarrhythmic therapies [9]. For both Sotalol and Dofetilide, for example, current recommendations include 3 days of inpatient hospitalization when initiating therapy, corresponding to the administration of 5 drug doses [10]. The requirement for inpatient monitoring during drug loading requires significant resources that strain overburdened healthcare systems. The ability to effectively monitor QT intervals in the outpatient setting represents an unmet need that would alleviate the need for hospital admission and monitoring during drug initiation.

The advent of wearable and pocket ECG monitoring systems raises the possibility of outpatient drug loading with monitoring for QT prolongation in carefully selected patients [11,12]. Central to the success of this approach is a reliable method for estimating QT intervals from ambulatory ECG recordings. Although manual review of ECG recordings by experienced health care providers remains the gold-standard for estimating QT intervals, this approach is limited by the availability of skilled clinicians who can review ambulatory ECG recordings from wearable and pocket ECG devices in a timely manner [11–14]. Inspired by the success of deep learning approaches for predicting a variety of clinically meaningful outcomes from the 12-lead electrocardiogram, several investigators have developed methods for automated QT-interval estimation using wearable or pocket ECG monitors [15,16]. For example, in one study a deep learning algorithm was designed to predict cardiologist over-read QTc values from a pocket ECG monitoring system. That study uses a Residual Neural Network architecture built on 2D convolutions that takes input of two ECG beats (extracted using proprietary algorithms from Lead-I and Lead-II ECG streams) and assigns each input to a QT range as a multiclass classifier. The ability of the method to predict QTc prolongation was evaluated in 686 patients with genetic heart disease–where half of these patients had long QT syndrome–and QTc prolongation was defined as a QTc>500ms [15]. Whereas the overall discriminatory ability of the method was excellent (AUC 0.97) in this small cohort, the ability of the model to detect clinically meaningful changes in the QTc was not evaluated. Case in point, for some medications, the threshold to either discontinue drug loading, or change the dosage, corresponds to a 15% increase in the QT or the QTc from baseline after drug administration, which can happen when the QTc<500ms [17]. Hence, an algorithm designed to only detect when the QTc>500ms cannot capture all clinically meaningful occurrences of QT prolongation. In another study, an artificial intelligence algorithm (AI-QTc) designed to be used with a smartwatch single-lead ECG, was applied to 85 patients with Covid-19 who were receiving hydroxychloroquine-azithromycin [16]. This study also uses convolutional neural network with residual blocks to classify the samples of an ECG as onsets and offsets of the P, QRS, and T waves and quantify QT interval accordingly. Overall, the agreement between predicted QTc intervals arising from the single-lead ECG and cardiologist over-read values from the 12-lead ECG was modest at best (errors greater than 40ms in a significant number of patients) and, more importantly, the ability of the method to detect clinically meaningful QTc prolongation over time was not evaluated.

The primary objective of this paper is to develop a solution for monitoring the QT prolongation risks from Lead-I ECG only for patients undergoing antiarrhythmic drug intake. Toward that goal, we propose a deep learning method that infers QTc intervals from ECG Lead-I—the lead that is ubiquitously available and easily acquired using a wearable or pocket ECG device, in contrast to the 12-lead clinical ECG [18]. Our proposed model, QTNet, demonstrates that the potential utility of such model is not limited to continuous QT monitoring, rather we can develop an alarm system based on our algorithm that identifies QT prolongation during Dofetilide loading. The ability to identify episodes of QT-prolongation using a single-lead ECG would enable outpatient monitoring for drug-induced QT prolongation and using wearable devices. The potential impact of ambulatory QT interval monitoring is significant, as it can reduce the healthcare burden associated with inpatient monitoring during the initiation of antiarrhythmic therapy. Moreover, outpatient QT interval monitoring would enable healthcare providers to assess the effect of different drug combinations on the QT interval over time.

## Methods

### Ethics statement

Retrospective analyses for this study were approved by the Institutional Review Board (IRB) at the Mass General Brigham (protocol #2020P000132). Given the analyses are conducted on retrospectively collected data, no consent was required or mandated by the IRB.

### Data acquisition

We used three datasets to develop and evaluate the model. The first dataset, which we refer to as the MGH-dataset, contains 4,223,689 12-lead ECG recordings, from 903,593 patients at the Massachusetts General Hospital (MGH), acquired between January 1981 and December 2020. PR, QRS and QT intervals for each ECG are also stored with each 12-lead ECG in the dataset, which were automatically measured by the ECG acquisition machine (mostly from GE and Philips). All machine generated measurements were included in the metadata of the ECG that were signed off by cardiologists, who often verified the measurements and interpretations if seemed necessary. The second dataset, which we refer to as the BWH-dataset, contains 3,171,283 ECG recordings from 667,060 patients who were seen at the Brigham & Women’s Hospital (BWH). The ECG and the interval labels have similar acquisition characteristics, similar clinical equipment was used in collecting those data. The third dataset is a publicly available databank, which we refer to ECGRDVQ-dataset from Physionet, and contains ECGs from 22 healthy subjects during anti-arrhythmic drug loading in a randomized, double-blind, 5-period crossover trial designed to compare the effects of QT prolonging drugs (i.e., Ranolazine, Dofetilide, Verapamil, and Quinidine) versus placebo on electrophysiological parameters (see https://physionet.org/content/ecgrdvq/1.0.0) [19,20]. The labels for QT interval and heart rate on this dataset were manually verified by a clinical expert ECG reader, which guarantees the reliability of the labels for the QT prolongation application.

### QTNet training and evaluation

**Fig 1:**
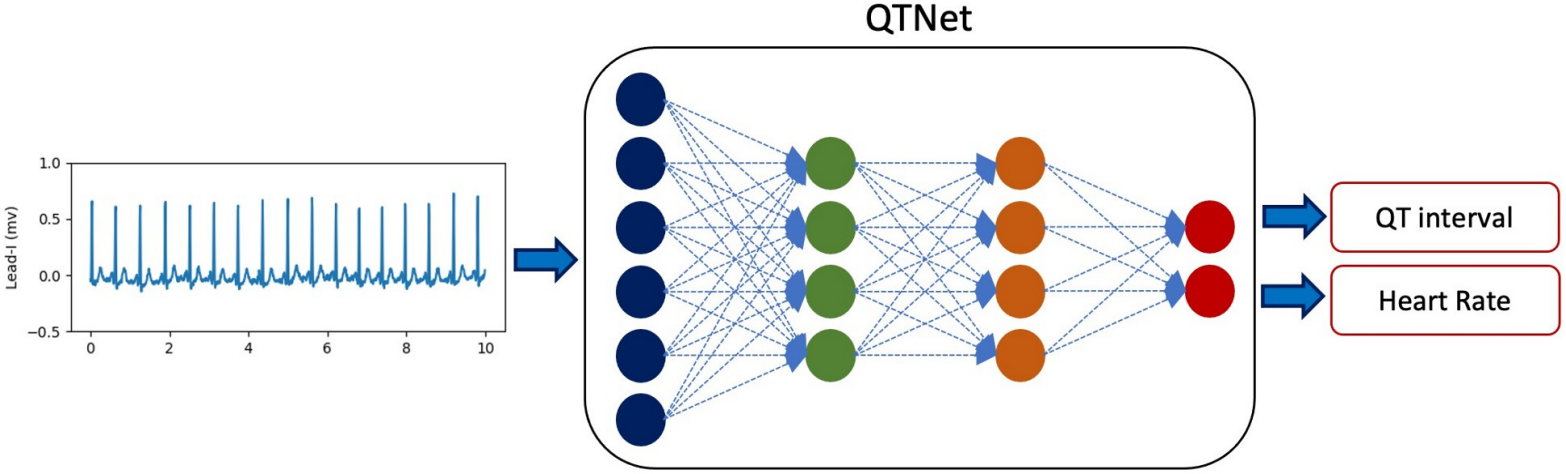
QTNet regression model. The model is trained on Lead-I 10-second ECG signals to estimate both the QT intervals and the heart rates.

QTNet is a deep learning model that infers QT intervals and heart rates from 10-second Lead-I ECG signals [Fig 1]. QTNet is built as a multi-task regression Convolutional Neural Network model based on a ResNet-18 architecture (see S1 File for more details on the regression model architecture). It is customized for single-lead ECG using 1-d convolution as the first layer of the architecture. The last layers of the model correspond to a multi-layer perceptron which regresses two quantities. The final output of the model is the QT interval in milliseconds and the heart rate in beats per minute. Data from the MGH-dataset were used to train and test the model for estimating QT interval and heart rate from Lead-I ECG. And data from the BWH-dataset were used to validate model results on a population distinct from the training set. Lastly, the ECGRDVQ-dataset was used to test whether the model could identify clinically meaningful QT prolongation during Dofetilide loading in a continuous monitoring setting.

In detail, the *Training* set contains 70% of the MGH-dataset (3.06 million ECGs, arising from 653 thousand patients). A development set or *Dev* set is used to determine when training should end and corresponds to 15% of MGH-dataset (534 thousand ECGs from 115.2 thousand patients), and the remaining15% of the data comprises our *Internal-test* set, which is used to test the model’s performance (633 thousand ECGs from 135.5 thousand patients). Given that each patient general has several ECGs, we ensured that all ECGs from a given patient only appeared in one of these three datasets, i.e., the datasets have no overlap with respect to patient data. Moreover, although ECGs in each of these datasets contain all 12 leads, only data from Lead-I is used to train and evaluate model performance.

We use a simple preprocessing pipeline in the model’s data loader to achieve fast training cycles and generalizability. First, we resampled the ECG signal to 250 Hz to match the model architecture. Then, we adjusted the baseline of the signal with a bandpass filter with passband between 0.05 Hz to 40 Hz, which also took care of high-frequency noise and artifacts. After this stage, any 10-second ECG which had an absolute voltage magnitude greater than 5 millivolts were excluded from the batch and were not part of model training and validation. We intentionally avoid any signal normalization on the Lead-I signal to preserve the relative amplitude information.

For training, initial model weights were set according to the Kaiming initialization method with random variables from a normal distribution with variance depending on the layer size [21]. Training involves minimizing an objective cost function, which in this case corresponds to the mean square error (MSE) between the predicted QT intervals and heart rates and their true values. For training, QT intervals and heart rates are normalized to zero-mean and unit variance distributions (i.e., Z-scored). Similarly, post-processing involved the reverse operation on model estimations to acquire the absolute values using the mean and the variance from the *Training* set. Back-propagation with an ADAM optimizer was used to minimize the cost function. For controlling the learning rate, we use a step scheduler to decay the rate in half every 3 epochs, starting from 0.01, and a batch size of 512 was used. Early-stopping was used to reduce risk of overfitting risk based on the validation loss. Essentially, training ends when the MSE starts to rise in the *Dev* set.

In addition to the *Internal-test* set, we evaluated the model on an *External-validation* set that contains 3.1 million ECG from 667 thousand patients in the BWH-dataset. For error between the estimated QT intervals and the interval labels, we calculate mean absolute error (MAE). These errors quantify the absolute difference between an individual estimation and its corresponding label and summarize over all estimations as the mean of those differences. Pearson’s correlation coefficient (Pearson-R) is used for assessing the amount of similarity between the estimated values and the labels.

### QT prolongation prediction-based alarm system

We used the ECGRDVQ-dataset, which contains ECG recordings from 22 healthy subjects who participated in a randomized, double-blind, 5-period crossover trial designed to compare the effects of QT prolonging drugs (i.e., Ranolazine, Dofetilide, Verapamil, and Quinidine) versus placebo on electrophysiological parameters (see https://physionet.org/content/ecgrdvq/1.0.0) [19]. For a given subject, three 12-lead ECGs were recorded 30 minutes before drug administration and again at 15 half-hourly/hourly intervals post-administration, and all QT intervals for each 12-lead ECG in this dataset were adjudicated by the same ECG reader [19].

**Fig 2:**
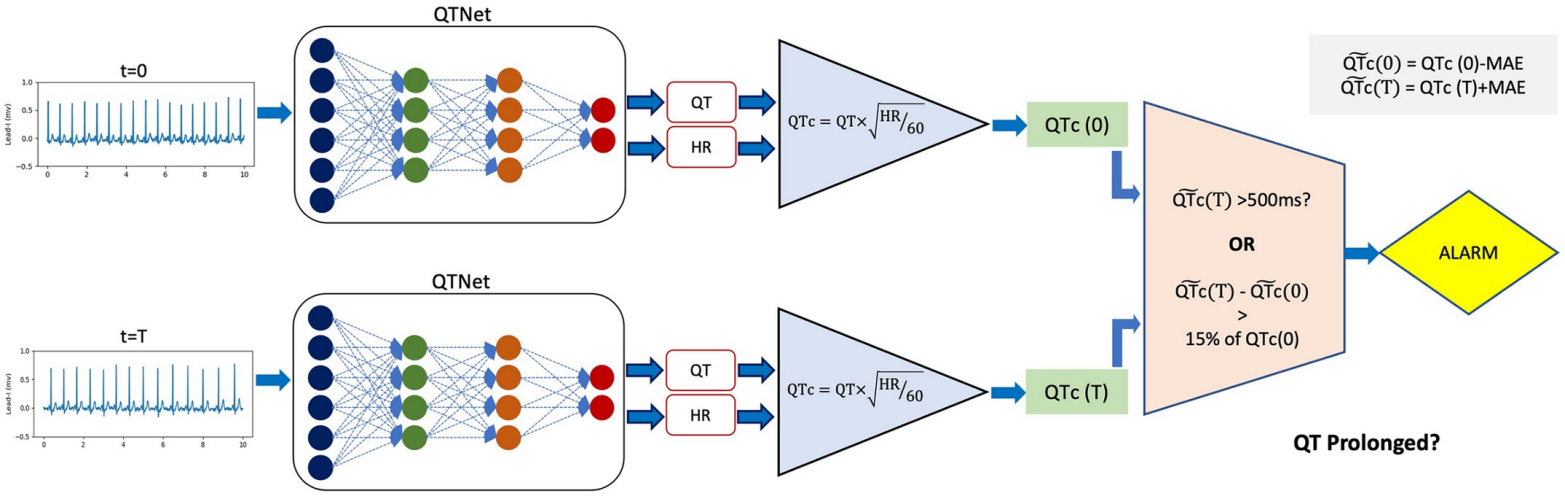
QT prolongation detection from QTNet. At any time t, QTNet is used to estimate the QT interval and the heart rate, which are then used to calculate the corrected QT interval using Bazett’s formula and adjust for the distribution shift using *Training*-MAE. Half-hour before drug loading (here, t = 0), we calculate adjusted QTc(0). At regular intervals t = T after the drug administration, the QTc(T) is calculated and adjusted, and used to detect possible prolongation. The detection is triggered if the adjusted value is greater than 500ms, or, if QTc(T) is increased by 15% compared to the pre-dosing adjusted interval QTc(0).

We use the procedure outlined in Fig 2 to identify clinically meaningful QTc prolongation events. The procedure uses both QTNet output values, as well as the expected errors in the predictions, which is obtained from the training data. We compute the QTc using Bazett’s formula [17,22]:

$$\text{QTc}=\text{QT}\;\times \sqrt{\text{HR}\;(\text{bpm})/60\;(\text{s}/\text{min})}$$

where the QT interval and heart rate are obtained as outputs from QTNet. We further define a clinically meaningful instance of QTc prolongation is defined as either:

1. The absolute QTc interval is longer than 500 milliseconds (ms),
2. The QTc interval is 15% longer than that the baseline value, which corresponds to the QTc before the administration of any drug.

At any time t, QTNet is used to estimate the QT interval and the heart rate, which are then used to calculate the corrected QT interval using Bazett’s formula and adjust for the distribution shift using *Training*-MAE. Half-hour before drug loading (here, t = 0), we calculate adjusted QTc(0). At regular intervals t = T after the drug administration, the QTc(T) is calculated and adjusted, and used to detect possible prolongation. The detection is triggered if the adjusted value is greater than 500ms, or, if QTc(T) is increased by 15% compared to the pre-dosing adjusted interval QTc(0).

For this study we rely on data corresponding to Dofetilide administration as this is the only medication used in this trial that requires inpatient monitoring during drug loading, and the above definition is consistent with current guidelines for loading several antiarrhythmic medications, include Dofetilide [17].

### Evaluation metrics for QT prolongation detection

Using the gold-standard, physician adjudicated QTc values, we calculated the sensitivity and specific of our algorithm for identifying clinically meaningful instances of QTc prolongation. Sensitivity values are calculated as the true positive rate–the rate of correct identification of QTc prolongation, and specificity as the true negative rate. For a given sensitivity, specificity, and prevalence of QT prolongation, the positive predictive value (PPV) and negative predictive value (NPV) are computed as follows:

$$\text{PPV}=\frac{\text{Sensitivity}\times \text{Prevalence}}{\text{Sensitivity}\times \text{Prevalence}+\left(1-\text{Specificity}\right)\times \left(1-\text{Prevalence}\right)}$$ (1)

$$\text{NPV}=\frac{\text{Specificity}\times (1-\text{Prevalence})}{(1-\text{Sensitivity})\times \text{Prevalence}+\text{Specificity}\times \left(1-\text{Prevalence}\right)}$$ (2)

where “Prevalence” refers to the prevalence of QTc prolongation instances in a given population. Eqs (1) and (2) are used to calculate the predictive values (PPV and NPV) for varying prevalence which corresponds to pre-test probability.

### QTc estimation with a Baseline Method

To compare the performance of the QTNet in both QT interval regression and prolongation detection, we used a baseline ECG delineation algorithm for QT interval and heart rate estimation as implemented in the NeuroKit library in Python, which is available via an open-source repository on GitHub (see https://github.com/neuropsychology/NeuroKit) [23,24]. Neurokit processing involves noise removal using a high-pass Butterworth filter followed by identification of R-peaks in a 10-sec single-lead ECG signal. Once the positions of R waves are known, the relative locations for the Q, P, S, and T waves using the slope of the ECG signal in predefined areas of each beat. The result is automated detection of Q wave peak and the T wave offset (end of T-wave).

## Results

QTNet estimates the QT interval and the heart rate corresponding using ECG Lead-1. Three datasets were used to train and evaluate the model [Table 1]. The Training and Internal-test datasets correspond to in-house registries of ECGs obtained from patients who receive their care at Massachusetts General Hospital (MGH) and the External-validation set contains patients who receive care at the Brigham and Women’s Hospital (BWH). The ECGRDVQ-dataset was used to assess the model’s ability to detect clinically meaningful QT prolongation during Dofetilide loading. As the latter dataset is comprised of healthy subjects who were enrolled in a randomized trial–as opposed to patients who are drug loaded to treat an underlying arrhythmia–this population is, on average, younger and have a lower resting heart rate relative to the MGH and BWH datasets.

**Table 1:** Demographics of patient population and other descriptions across the three datasets.

Dataset	Training	Internal-test	External-validation	ECGRDVQ
Number of Patients	652,845	135,539	667,060	22
Number of ECGs	3,056,660	632,997	3,171,283	5232
Age (yr)	60.6 ± 18.7	60.4 ± 18.7	60.3 ± 16.4	27 ± 5.4
Female (%)	42.8	43.3	50.2	49.5
HR (bpm)	77 ± 20.3	77 ± 20.2	77 ± 18.7	64 ± 9.5
QT (ms)	394 ± 50.0	394 ± 49.8	396 ± 47.6	400 ± 33.7
QTc (ms)	438 ± 38.5	438 ± 38.3	440 ± 35.3	412 ± 36.2

### Estimating QT intervals with QTNet

A defining characteristic of QTNet is that it estimates the QTc only using data from Lead-I, i.e., the ECG lead most often acquired by pocket and wearable ECG monitoring devices [11–14]. Moreover, instead of directly predicting the QTc using a pre-specified formula, QTNet estimates the absolute QT interval in milliseconds in addition to the average heart rate, where both quantities are inferred using data from Lead-I alone [Fig 1]. With these data, the QTc can be calculated using a variety of existing methods, e.g., Bazett, Framingham, Fridericia, and Hodges formulas [22,25–27].

**Fig 3:**
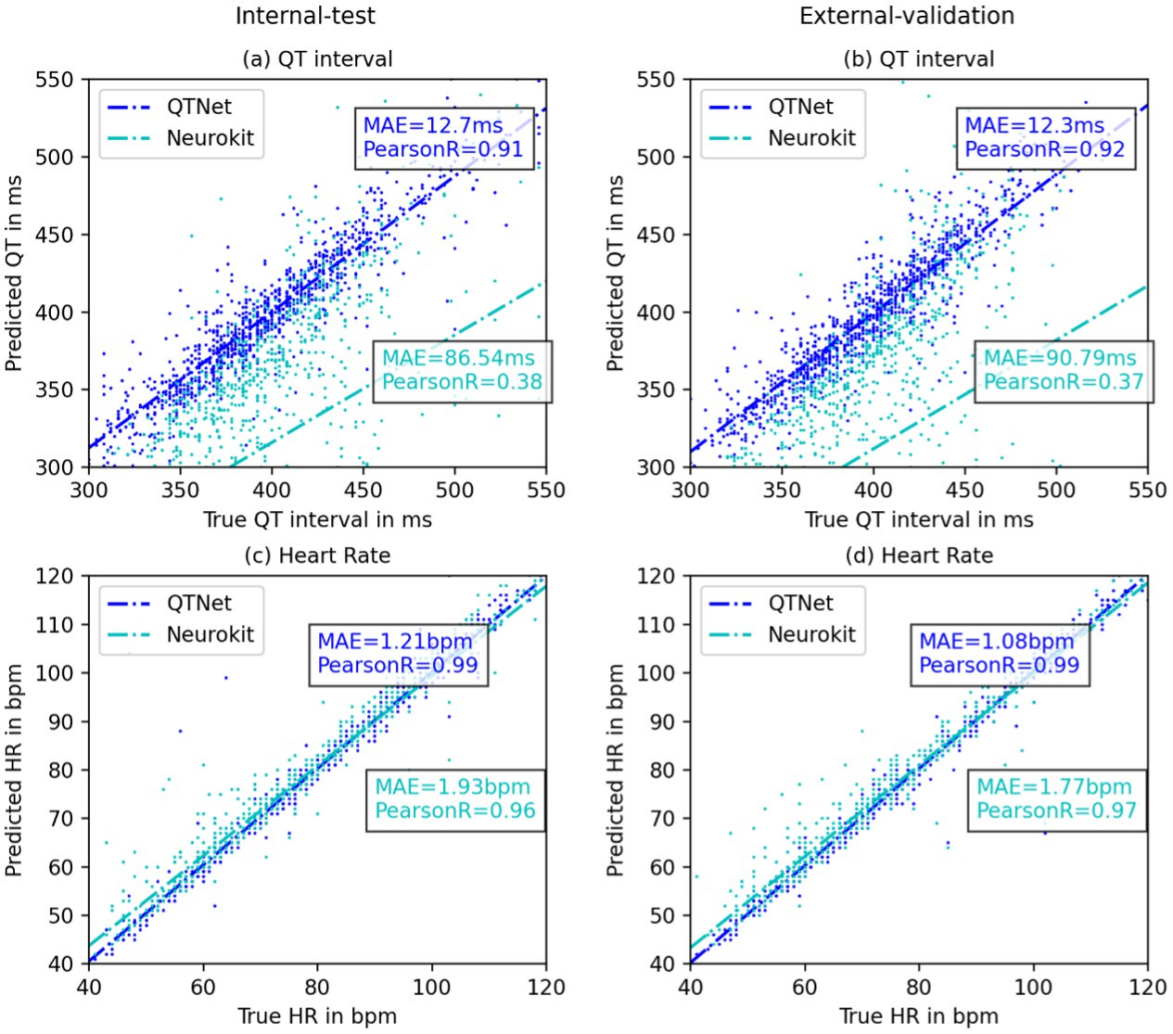
QTNet regression performance on test data. The estimated QT intervals and HR are compared with the corresponding labels on two datasets: *Internal-test* (MGH test) and *External-validation* (BWH) using two metrics of performance: mean absolute error (MAE) and Pearson-R correlation coefficient. The plots show the comparative performance of QTNet with the baseline algorithm implemented using Neurokit2 library. (a) The estimation errors for QT estimation using QTNet are very low (about 12 milliseconds) and the correlation coefficients are very high (0.91) on the *Internal-test* set, and (b) remains steady even to *External-validation* set which was not seen by QTNet during training. (c) and (d) show notable performances in HR estimation both by QTNet and the Neurokit algorithm across datasets.

The performance of QTNet with respect to estimating QT intervals and average heart rates is shown in Fig 3. For both the Internal-test set and the External-validation set, the overall predictive performance for both heart rate and QT interval estimation is excellent, with mean absolute errors (MAE) approximately 12ms for absolute QT estimates and 1.2 bpm for heart rate estimates. Corresponding Pearson correlation coefficients are 0.91 for QT estimates, and 0.99 for heart rate estimates [Fig 3]. For comparison, we also tested an established automated method for estimating ECG intervals and heart rates, as implemented in the NeuroKit library [23]. That algorithm was performing comparably for heart rate estimation with an MAE of 2 bpm and Pearson coefficient of 0.96 in the Internal-test set and an MAE of 1.8 bpm and correlation coefficient of 0.97 in the External-validation set. But the estimations of QT intervals were relatively poor; the MAE for QT internals using NeuroKit was 86.5 ms in the Internal-test set and 90.8 ms in the External-validation set with Pearson correlation coefficients of 0.38 and 0.37, respectively [Fig 3].

### Detecting Drug-induced QT Prolongation

QTNet estimates QT intervals and heartrates (HR) from 10-second Lead-I ECG signals, which are used to assess whether QT is prolonged at any instance. The QTc is calculated using the estimated QT interval and HR, then adjusted using the mean absolute error (MAE) achieved on the training dataset. The QTc estimated from an ECG taken before the drug administration is used as the reference value for any other QTc estimated after the drug is administered. QT prolongation is detected at any time after dosing, using a QTNet based alarm system, i.e., either the QTNet estimated QTc>500ms or the QTNet estimate corresponds to a 15% increment from the pre-dosing reference QTc (see Fig 2 and Methods for details).

We used QTNet to identify instances of clinically meaningful QT prolongation in the ECGRDVQ-dataset. This dataset contains ECG data from subjects both before and after Dofetilide dosing. The discriminative performance of QTNet in identifying the post-administration instances when QT prolongation occurred is shown in Table 2. For comparison, we also list results obtained using the QT interval estimation method in NeuroKit. QTNet achieves 87% sensitivity and 77% specificity and an overall accuracy of 80% for detecting when QT prolongation occurred. These values are considerably larger than those obtained with NeuroKit.

**Table 2:** Detecting QT prolongation instances after Dofetilide dosing on the ECGRDVQ dataset; the prevalence for the prolongated QT interval is 25% from the 330 temporal instances.

Methods	NeuroKit	QTNet
Sensitivity	0.29 ± 0.10	0.87 ± 0.07
Specificity	0.83 ± 0.05	0.77 ± 0.05
Accuracy	0.70 ± 0.05	0.80 ± 0.04

**Fig 4:**
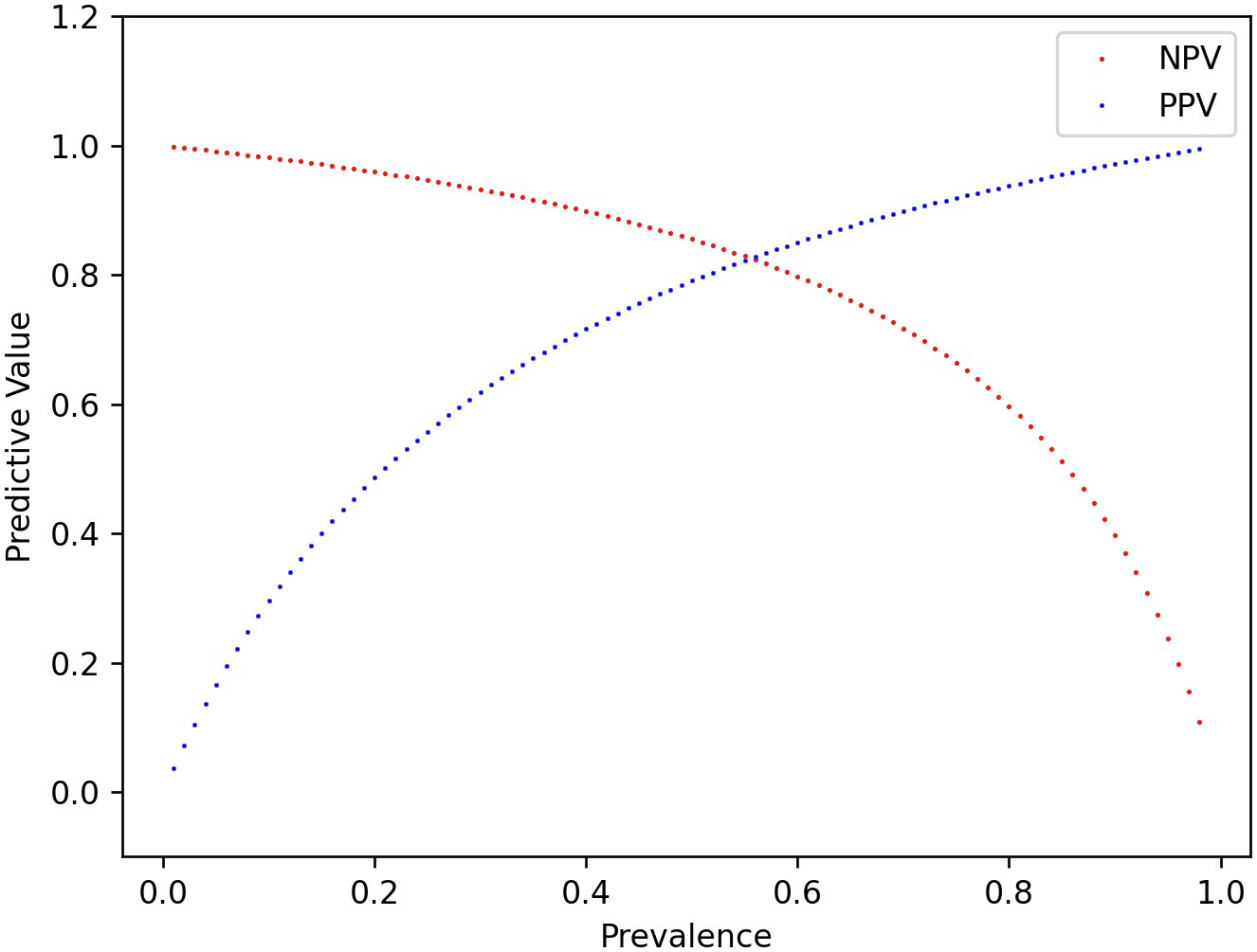
Predictive values of QTNet in detecting QT prolongation. The RDVQ dataset contains 24 hours data (N = 330) from 22 subjects. In 25% of those instances, mostly within 3 hours of Dofetilide administration, the QT prolongation was observed. QTNet can detect 80% of those instances with 87% sensitivity and 77% specificity. Using Eqs (1) and (2), the negative and positive predictive values (NPV and PPV) are plotted for varying prevalence.

To gauge how the model can be used in practice, we calculated the positive predictive value (PPV) and negative predictive value (NPV) for QTNet detecting drug-induced QT prolongation events. Both the PPV and the NPV can be calculated from the sensitivity, specificity and the prevalence of the drug-induced prolongation events, as outlined in Eqs (1) and (2) in the Methods section. The resulting PPV is 56% and the NPV is 95%. More generally, Fig 4 plots the PPV and NPV as a function of the population prevalence and therefore depicts model predictive performance in populations with different intrinsic rates of QT prolongation.

**Fig 5:**
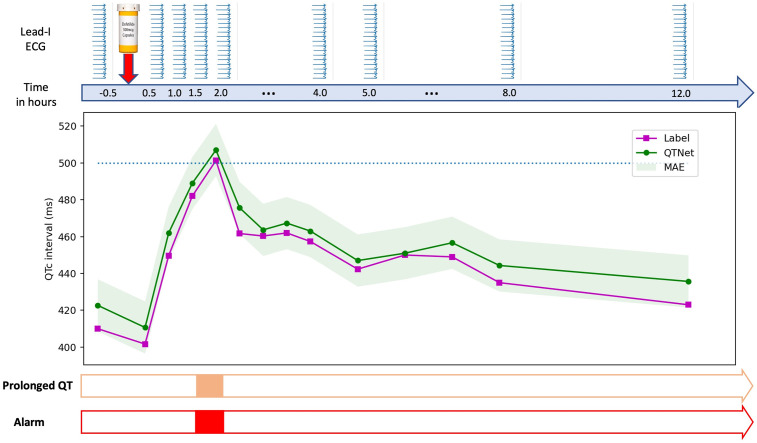
QTNet tracks the QT prolongation risk. In this example plot, Dofetilide 500ug was administered at time t = 0 hour for a patient, and the estimated values of QT interval and the corresponding labels are shown across time. The estimations by QTNet follow the labels within one MAE prediction bounds. QT prolongation instances were detected at t = 1.5 and t = 2 comparing the estimations at those instances with the pre-dosing estimation (t = -0.5 hour). The predictions and the labels for the QT prolongation overlap at t = 1.5 and t = 2 (1.5 and 2 hours after administration).

An example timeseries of the QTc values for a subject from pre-dosing to 12 hours after administration are shown in Fig 5. In this example plot, Dofetilide 500ug was administered at time t = 0 hour for a patient, and the estimated values of QT interval and the corresponding labels are shown across time. The estimations by QTNet follow the labels within one MAE prediction bounds. QT prolongation instances were detected at t = 1.5 and t = 2 comparing the estimations at those instances with the pre-dosing estimation (t = -0.5 hour). The predictions and the labels for the QT prolongation overlap at t = 1.5 and t = 2 (1.5 and 2 hours after administration).

## Discussion

Inpatient administration and monitoring during drug loading remain a mainstay of clinical practice for a number of antiarrhythmic therapies. Since the purpose of such monitoring is mainly geared towards identifying significant episodes of QT prolongation, we explored whether a machine learning algorithm can be used to estimate QT intervals in an automated fashion and identify episodes of drug-induced QT prolongation. Furthermore, as our goal is to develop a method that can leverage information obtained from wearable and pocket-held devices, we developed a deep-learning algorithm, QTNet, that only uses data from ECG Lead-I, i.e., the lead that is commonly acquired in outpatient ECG monitoring devices. The novel contributions of our approach are not only limited to developing a method that estimates QT intervals from a single-lead ECG but also to test the method’s ability to detect clinically meaningful QT prolongation for continuous patient monitoring. The novel contributions of our study are the development of a deep learning algorithm that estimates QT intervals from a single-lead ECG and the development of a method, based on the algorithm’s output, for detecting clinically meaningful QT prolongation events. Unlike prior studies, we consider clinically meaningful criteria for identifying prolonged QT intervals. Indeed, a QTc greater than 500ms or a 15% increase in the QTc (using Bazett’s formula) from baseline after Dofetilide administration warrants a decrease in dosage or discontinuation of Dofetilide. Our approach explicitly considers both of these criteria when identifying QT prolongation events.

Clinically, QT interval measurements arise from either automated machine reads using proprietary algorithms that rely on a linear combination of QT readings from multiple leads–typically the mean of all 12 QT intervals–or from manual physician measurements [28,29]. Manual physician reads are often made using either Lead-II or one of the precordial leads [28]. The upshot being that ECG Lead-I alone is not typically used for measuring the QT interval in clinical settings. Nevertheless, QTNet was trained to produce the same values that would arise from automated ECG reads that have been reviewed by a cardiologist. More importantly, QTNet identifies instances of drug-induced prolongation in the ECGRDVQ-dataset, where QT intervals are measured using Lead-II and verified by human ECG readers. Hence QTNet yields QT interval measurements using Lead-I that are similar information to what obtained from analyses of the 12-lead ECG.

We evaluated the method using three distinct datasets. In our internal and external-validation datasets, the performance by QTNet remains consistent with mean absolute errors of 12.63ms and 12.30, respectively. For comparison, the median absolute error between QTc measurements using Lead-I of the Apple Watch and the 12-lead ECG is 18ms [13]. We further demonstrate the QTNet can identify instances of Dofetilide-induced QT prolongation that were identified from an analysis of the corresponding 12-lead ECG. Most notably, the associated sensitivity and specificity for identifying drug-induced QT-prolongation was 0.87 and 0.77, respectively. The corresponding NPV was 95% in this cohort, which has an underlying prevalence of 25% for QT prolongation events.

ECG data from our dataset correspond to high-quality Lead-I ECG signals, rather than an ambulatory ECG monitor. This is important because the signal quality from wrist-worn ECG devices can be poor relative to the Lead-I ECG [30]. However, bipolar single-lead wearable ECG patch monitors, which are applied to the chest wall, yield signals comparable to what is obtained with multi-lead high-quality ECG devices [31,32]. Consequently, our results are likely more applicable to data obtained with a patch monitor applied to the chest, rather than a smart watch acquired ECG signal.

Patients who are deemed to be at high risk of drug-induced QT prolongation (e.g., individuals with an elevated QTc at baseline or who are already taking medications associated with QT-prolongation, etc.) benefit from inpatient drug loading—in keeping with current guidelines. However, it is not clear whether low risk patients (e.g., normal QTc at baseline, no concomitant use of QT-prolonging drugs, no family history of QT-prolongation, etc.), derive the same benefit. Indeed, outpatient drug-load with contemporaneous QT monitoring via a wearable or pocket ECG monitoring device may be viable mechanism for patients who have a low pre-test probability of drug-induced QT-prolongation. Our results suggest that QTNet has a high NPV for identifying instances of QT-prolongation when the underlying population has a low prevalence of QT-prolongation [Fig 4]. As patients who are deemed to be low risk, by definition, comprise a population with a low prevalence for QT-prolongation events, the NPV of QTNet is expected to be similarly large when the pre-test probability is low. Therefore, a negative prediction in patients who have a low pre-test probability for drug-induced QT-prolongation strongly suggests that clinically significant QT prolongation is not present, and that drug dosing can continue without modifying the drug dose.

Our study has limitations. As we do not explicitly use data arising from such wearable devices, our approach requires further prospective validation in an ambulatory setting. QTNet associated sensitivity and specificity for identifying instances of clinically meaningful QTc prolongation events were calculated from a population of healthy individuals who were given Dofetilide. Further prospective validation in low-risk clinical cohorts is warranted. In particular, the positive predictive value of our alarm system is expected to be reduced in populations that have low prevalence for drug-induced QTc prolongation. In addition, as we did not have access to demographic information of the patients in our study, we are unable to determine how QTNet performance varies as a function of ethnicity, race, and age. Such information is necessary to understand algorithmic bias–if any–and model performance in clinical cohorts that are traditionally under-represented in clinical studies. Additional studies are therefore needed to fully explore the utility of QTNet in different patient subgroups, and we envision such studies to address the challenges ranging from real-world patient monitoring feasibilities to better representation of the underlying relationship and clinical study design.

## Conclusions

The QT interval and heart rate can be accurately estimated from a single-lead ECG using deep learning. Clinically meaningful instances of QT prolongation can further be identified using a single-lead, thereby enabling outpatient monitoring for QT prolongation in patients who have a low-pretest probability for drug-induced QT prolongation.

## Supporting information

S1 File QTNet Model Description. (DOCX)
