# Supplementary material for: Detecting QT prolongation from a single-lead ECG with deep learning
Source: PLOS Digit Health. 2024 Jun 25;3(6):e0000539. doi: 10.1371/journal.pdig.0000539 (PMC11198807; doi:10.1371/journal.pdig.0000539)
Supplement: S1 File — (DOCX) [file pdig.0000539.s001.docx]

**QTNet Model Description**

QTNet is a single-channel residual neural network (ResNet-18) consis­ting of four residual blocks. Each residual block comprised of two convolutional layers and a skip connection. We add a 1-d convolution layer before the residual blocks to ingest the 10-second Lead-I ECG signal as a single-channel input. The representation from the residual blocks is passed on to two fully-connected dense layers that outputs two random variables as the estimate of QT interval and heart rate, respectively, and thus completes the end-to-end regression architecture [Fig A].

We used a kernel size of 16 units for all convolutional filters. The input channel is of 1x2500 samples length, as we resample all 10-second ECG Lead-I signals to 250 Hz sampling rate to get the input tensor. The ingest layer convolves this tensor with single sample stride over 64 filters. We learn convolu­tion layers with 128, 196, 256, and 320 filters for the four consecutive residual blocks. For each block, the skip connections are implemented with max pooling and 1-to-1 convolution layer. Batch normalization and ReLU activation layers follow each convolution layer of the model. Using average pooling, we get a 1x320 feature representation from the output of the last residual block. Two fully-connec­ted dense layers, connected in tandem, learn the weights to regress two outputs from this 320-d representation: QT interval and heart rate. We use PyTorch framework to implement the architecture as well as for training and evaluation.

*Fig A*: **QTNet Architecture**. QTNet is designed as single-channel Resnet-18 multi-output regression pipeline. The model takes in a 10-second Lead-I ECG signal and estimates both the QT interval and the heart-rate corresponding to that ECG.
